# Supplementary material for: MetaRibo-Seq measures translation in microbiomes
Source: Nat Commun. 2020 Jun 29;11:3268. doi: 10.1038/s41467-020-17081-z (PMC7324362; doi:10.1038/s41467-020-17081-z)
Supplement: Supplementary file 10 — Supplementary Data 7 [file 41467_2020_17081_MOESM10_ESM.zip › File2/Confidence_High_Taxonomy/190475_out.krona.html]

Javascript must be enabled to view this page.

members
magnitude
magnitudeUnassigned
count
unassigned
taxon
rank

190475\_out

2

2
superkingdom
2

2
1239
phylum

186801
class
2

2
186802
order

186803
family
2

572511
genus
2

species
1262757

SRS075878\_contig\_number\_14897SRS144537\_contig\_number\_42795
2
